# Multidrug-resistant organism (MDRO) contamination of nursing home staff hands and forearms when entering the breakroom or going home

**DOI:** 10.1017/ice.2026.10477

**Published:** 2026-05-28

**Authors:** Kevin Nguyen, Cassiana E. Bittencourt, Gabrielle M. Gussin, Julie A. Shimabukuro, Raveena D. Singh, Raheeb Saavedra, Susan S. Huang

**Affiliations:** 1 Fielding School of Public Health, University of California Los Angeles, Los Angeles, CA, USA; 2 Division of Infectious Diseases, University of California Irvine School of Medicine, Irvine, CA, USA; 3 Department of Pathology and Laboratory Medicine, University of California, Irvine School of Medicine, Irvine, CA, USA; 4 Epidemiology & Infection Prevention, University of California Irvine Health, Orange, CA, USA

## Abstract

Forearms and hands of 100 staff from five nursing homes were sampled on breakroom entry or end-of-shift. Twenty (20%) were multidrug-resistant organism (MDRO)-positive (90% methicillin-resistant *Staphylococcus aureus*). Recent high contact resident-care activities were associated with contamination (OR = 9.1 (1.1–74.2), *P* = .04). Enhanced barrier precautions and hand hygiene are important to prevent staff MDRO acquisition.

## Introduction

Up to 50%–65% of nursing home residents harbor an MDRO,^1, 2^ partly due to transmission within the nursing home.^3^ High contact care activities such as bathing, dressing, transferring, and toileting readily transfer multidrug-resistant organisms (MDROs) to staff gowns and gloves.^4^ While it is expected that staff perform hand hygiene before and after contacting residents or their environment, and also expected that enhanced barrier precautions (EBP) with gowns and gloves accompany high contact activities in high-risk individuals, adherence is often poor.^5^ Staff adherence with personal protective equipment (PPE) has been tied to their perceived risk from care activities.^6^ Moreover, the COVID-19 pandemic demonstrated that staff were more adherent when the perceived risk was to themselves versus their risk of spreading contagion to others.^7^ For this reason, we evaluated the likelihood of staff harboring MDROs on their hands and forearms when entering the breakroom to eat or when leaving to go home at the end of their shift.

## Methods

We conducted a cross-sectional observational study of MDRO contamination of staff hands and forearms in five nursing homes during December 2024 in Orange County, California. Staff were approached on breakroom entry during lunch, or facility exit at end-of-shift to achieve 20 volunteer participants per nursing home. Care was taken to avoid behavioral contamination by ensuring an unannounced single visit of short duration (1–2 h). Participants were also instructed to not inform other staff about the study for 2 hours postparticipation. A $10 token gift card was provided for participating in the 10-minute study. This study was approved by the University of California Irvine (UCI) Institutional Review Board.

Participants were administered a brief structured survey related to role, care activities in the past two hours, and glove use during those activities. Resident care activities were categorized as bathing/showering, dressing, turning/transferring, toileting, wound/device care, feeding, activity monitoring, administering medications, and non-clinical. Glove use was recorded as “always,” “sometimes,” or “never” for overall activities in the past two hours.

Bilateral hands (palm, dorsum), wrists, and forearms were swabbed using a nylon-flocked swab (ESwab, Copan Diagnostics). If staff had covered forearms, swabs were taken of the overlying clothing. Samples were processed within 4 hours by the UCI clinical microbiology laboratory and cultured for methicillin-resistant *Staphylococcus aureus* (MRSA), vancomycin-resistant enterococci (VRE), extended-spectrum beta-lactamase producers (ESBL), carbapenem-resistant enterobacterales (CRE), carbapenem-resistant *Acinetobacter baumannii* (CRAB), and *Candidozyma auris*.

Swabs were swirled into 1 mL of liquid Amies, and 100µL were inoculated onto chromogenic agar for MRSA (Spectra MRSA, Remel), VRE (Spectra VRE, Remel), and *C. auris* (CHROMagar Candida, BBL). For ESBL, CRE, and CRAB, 100µL were inoculated onto MacConkey agar. ESBLs were initially screened using a cefpodoxime disk and confirmed by double disk diffusion using ceftotaxime and cefotaxime/clavulanic acid. CRE and CRAB were confirmed with a meropenem disk. All species were verified by matrix-assisted laser desorption/ionization time-of-flight mass spectrophotometry (MALDI-TOF).

The percent of staff with any hand/forearm MDRO contamination was plotted by staff type, resident care activities performed, and glove use. Staff were grouped into three categories: (1) certified nursing assistants (CNAs), (2) licensed/registered nurses (LVN/RN) and physical/occupational therapists (PT/OT), and (3) non-clinical staff. Care activities were grouped into high contact (bathing/showering, dressing, turning/transferring, toileting, or wound/device care),^4, 8^ and low contact (feeding, activity monitoring, administering medications, and non-clinical) activities. Bivariable and multivariable logistic regression models assessed factors associated with staff MDRO contamination (SAS/STAT 15.3 (SAS, Cary, NC).

## Results

**Figure 1.**
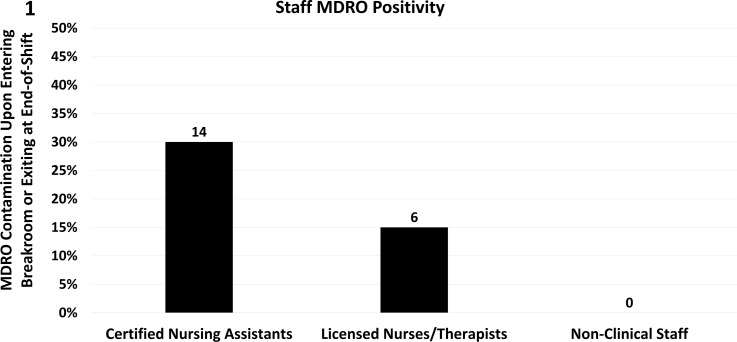
MDRO contamination of staff hands/forearms upon entering breakroom or exiting at end-of-shift. Graphic displaying the percentage of staff with MDRO-positive hand or forearm cultures upon entering the breakroom or leaving at end-of-shift, by staff type (certified nursing assistants (CNAs) or licensed nurses and therapists (includes registered nurses, licensed vocational/practical nurses, physical therapists, and occupational therapists). There was no MDRO contamination found among non-clinical staff (not shown).

Twenty staff participated per nursing home, for a total of 100 participants. These included 47 (47%) CNAs, 39 (39%) licensed nurses and therapists (7 RNs, 23 LVNs, 9 PT/OTs), and 14 (14%) non-clinical staff. Overall, 20 (20%) staff had hands/forearms contaminated with MDRO, including 18 (90%) with MRSA, 1 (5%) with ESBL, and 1 (5%) with CRAB. Figure 1 displays the distribution by staff type that had MDRO contamination upon entering the breakroom or leaving for the end-of-day shift.

Participants reported performing the following activities in the two hours prior to participation: turning/transferring (62%), dressing (43%), toileting (39%), bathing/showering (34%), activity monitoring (33%), feeding (30%), administering medications (25%), providing wound/device care (13%), and non-clinical (14%). Overall, a total of 292 care activities were reported with 74% of staff performing any high contact activity, and 26% performing only low or no contact activities.

**Figure 2.**
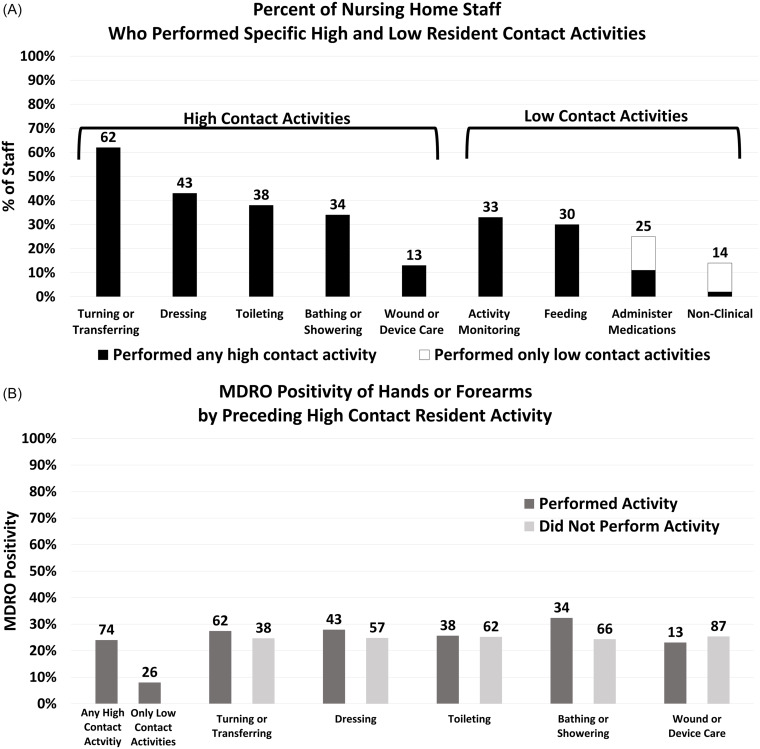
(A) percent of nursing home staff who performed specific high and low resident contact activities. (B) MDRO contamination of hands or forearms by preceding high contact resident activity. Graphic displaying percentage of nursing home staff (N = 100) performing various types of resident care activity in the past 2 hours (Panel 2A). High resident contact activity bars are indicated in black for that specific activity. For low resident contact activities, black is also used to indicate the portion of staff performing that specific low-contact activity who also perform any high resident contact activities. The white portion of the stacked bar indicates the portion of staff performing that specific low contact activity who did not perform any high resident contact activities. Panel 2B shows the percentage of staff that performed any high contact activities and only low contact activities as well as performance in any high contact resident care activities had higher percentages of MDRO contamination among healthcare staff, except for those doing wound or device care.

Figure 2 displays the distribution of staff hand/forearm MDRO contamination by resident care activity in the prior two hours. Among the 74 staff who performed any of the high contact activities, 18 (24%) had MDRO contamination. Among the 26 staff who performed only low or no contact activities, 2 (8%) had MDRO contamination.

Related to glove use in the two hours prior to sampling, 61 (61%) participants reported that they always wore gloves, 32 (32%) reported some use, and 7 (7%) reported no use. MDRO contamination was 12 (20%) among those who always wore gloves, 6 (19%) among those with some usage, and 2 (29%) among those who did not use gloves.

Bivariable analyses identified bathing and showering, turning and transferring, and feeding as associated with MDRO contamination. In multivariable models adjusting for high contact activities and glove use, only high contact activity was significantly associated with MDRO contamination (OR = 9.1 (CI: 1.1–74.2), *P* = .04). Staff type was excluded due to collinearity with activity type.

## Discussion

This evaluation of nursing home staff found that 20% had MDRO contamination of their hands and/or forearms upon entering the breakroom to eat or leaving for home at end-of-shift. These represent moments when staff expect themselves to be free of MDRO pathogens for personal safety. Contamination was markedly associated with high contact activities which readily transfer MDROs from resident carriers to staff.

While we evaluated six pathogens, staff contamination was predominantly due to MRSA even though resident prevalence of MRSA and ESBL are each 30%–50%, and VRE is 15%–20% in Southern California nursing homes.^1^ This is consistent with MRSA’s greater ability to readily colonize/contaminate, persist, shed, and spread from carriers and environmental objects.^9^

These findings could potentially be mitigated by placing hand hygiene dispensers at entry points to breakrooms and at facility exit points. Ideally, staff would hand wash prior to eating, but alcohol gel is preferable to no hand hygiene. Interestingly, gloves were not protective against HCW contamination, possibly because gloves do not protect against arm/body contact transmission, use is often imperfect or improper, and use was self-reported. Ensuring high adherence to EBP may be necessary to prevent staff MDRO contamination. However, whether EBP should be limited to high-risk residents with devices and known MDROs or applied to all high contact activity remains unclear given that 82% of MDRO carriage is unknown to the nursing home.^1^ Adopting universal decolonization can further reduce shedding of MDROs by residents onto caregivers, and decrease hand contamination among nurses.^10^ These results can be used to train staff on safe practices since PPE adherence increases when personal risk is perceived.^7^

Limitations include small sample size, regional focus, and convenience sampling, and self-reported activities. Hand hygiene before and after PPE use was not collected, limiting the scope of the study. It is also possible that more distant care activities were the source of MDRO contamination.

In summary, MDRO contamination of nursing home staff hands and forearms, particularly with MRSA, is common in breakrooms and when leaving to go home. Effort is needed to promote hand hygiene, EBP, and decolonization opportunities for staff safety.
